# Management skin manifestation of multisystem inflammatory syndrome associated with SARS-CoV-2

**DOI:** 10.1186/s12985-021-01736-4

**Published:** 2022-01-06

**Authors:** Yeter Eylul Bayram, Dilek Yildiz-Sevgi, Ayse Yavuz, Merve Cancetin, Mehmet Yavuz Gurler

**Affiliations:** 1 Department of Internal Medicine, Hamidiye Sisli Etfal Education and Research Hospital, Istanbul, Turkey; 2 Department of Infection, Hamidiye Sisli Etfal Education and Research Hospital, Huzur Mah. Cumhuriyet&Demokrasi Cad. No 1/3. Sariyer, Istanbul, Turkey

**Keywords:** COVID-19, SARS-CoV-2, A multisystem inflammatory syndrome, Dermatology

## Abstract

**Background:**

Multisystem inflammatory syndrome (MIS), which develops after a past covid-19 infection. MIS can be described in different tissue inflammation, including the heart, lung, kidney, brain, skin, eye, and or gastrointestinal organs at the presence of COVID-19. Initially, MIS was described in Europe in children infected with SARS-CoV-2, then it was recently seen in the USA in 2020. MIS is a rare but serious disease condition associated with COVID-19 that can affect children (MIS-C) and adults (MIS-A).

**Case presentation:**

A 44-year-old male who showed MIS-A in 59-day after his first covid-19 contact history. The patient presented to our emergency department with complaints of high fever, nausea, weakness, redness of the eyes, headache, and joint pain. On the second day of his hospitalization, a maculopapular skin lesion was seen in most of the skin. His fever could not be controlled even given paracetamol and broad effective antibiotics. His clinical, radiological, and laboratory findings showed that he had MIS-A. The patient was given intravenous pulse methylprednisolone and intravenous immunoglobulin (IVIG). These treatments, then, resulted in improvement of his clinical conditions, including fever and skin lesions, on the second day of the treatment. The patient was discharged in 14 days after the treatment.

**Conclusion:**

This report indicated that diagnosis and treatment of MIS-A could result in reducing patient morbidity and mortality.

## Background

The COVID-19 outbreak quickly spread around the world after the first cases were reported in China in December 2019. More than 182 million cases of COVID-19 caused by SARS-CoV-2 infection have been reported and more than 3.9 million deaths have occurred by July 2021 [1]. (SARS-CoV-2) can cause respiratory tract diseases of varying severity. It is generally milder in children than adults and a significant proportion of children with the disease are asymptomatic [2]

Recently, Post-COVID syndrome has been reported as a new clinical condition in the context of SARS-CoV-2, called multisystem inflammatory syndrome (MIS) [3]. It was initially defined by Greenhalgh in 2020 [4] and seen in children (MIS-C) [5] in April 2020. Recently, MIS has also reported in adults (called, MIS-A) [6]. Even MIS-C is initially defined in children, similar clinical characteristics have been shown in adult form (MIS-A) [7]. However, MIS-A shows some phenotypic differences from MIS-C (summarized in Table 1) [8]. The patients with MIS-A syndrome might complaint abdominal pain, redness of the eyes, chest pain, vomiting, diarrhea, fatigue, headache, and/or low blood pressure [7]. Generally, the CDC reported that MIS-A syndrome could be considered as the most likely diagnosis if patients met the following criteria in the case definition; a serious illness requiring hospitalization in a person aged ≥ 21 years; laboratory evidence of current or previous SARS-CoV-2 infection (positive PCR or antibody test) at the time of application or within the previous 12 weeks; severe dysfunction of one or more extrapulmonary organ systems (for example, hypotension or shock, cardiac dysfunction, arterial or venous thrombosis or thromboembolism or acute liver injury); laboratory evidence of severe inflammation (such as, elevated CRP, ferritin, D-dimer or IL-6); absence of severe respiratory disease (to exclude patients in whom inflammation and organ dysfunction can simply be attributed to tissue hypoxia) [2]. However, more data are needed to better understand the clinical symptoms, pathogenesis, risk factors, clinical-laboratory variables, and outcomes of the disease and treatment. We aimed to contribute to the case definition by presenting our report. We also wanted to draw attention to the fact that a proper diagnosis and rapid treatment of MIS-A can reduce the patient morbidity and mortality.

**Table 1:** Summarizing differences between MIS-A and MIS-C

Phenotypes	MIS-A	MIS-C
Prevalence	Less common	More Common
Myocarditis	54%	29%
Cardiac dysfunction	30%	15%
Arterial thrombosis, pulmonary embolism, and/or deep venous thrombosis	5%	1%
Dermatologic findings	46%	76%
Hospital stays	~ 8 days	~ 5 days
Ventilation	25%	9%
Sex	70% male	60% male
Mortality	7%	1%

## Case presentation

**Fig. 1:**
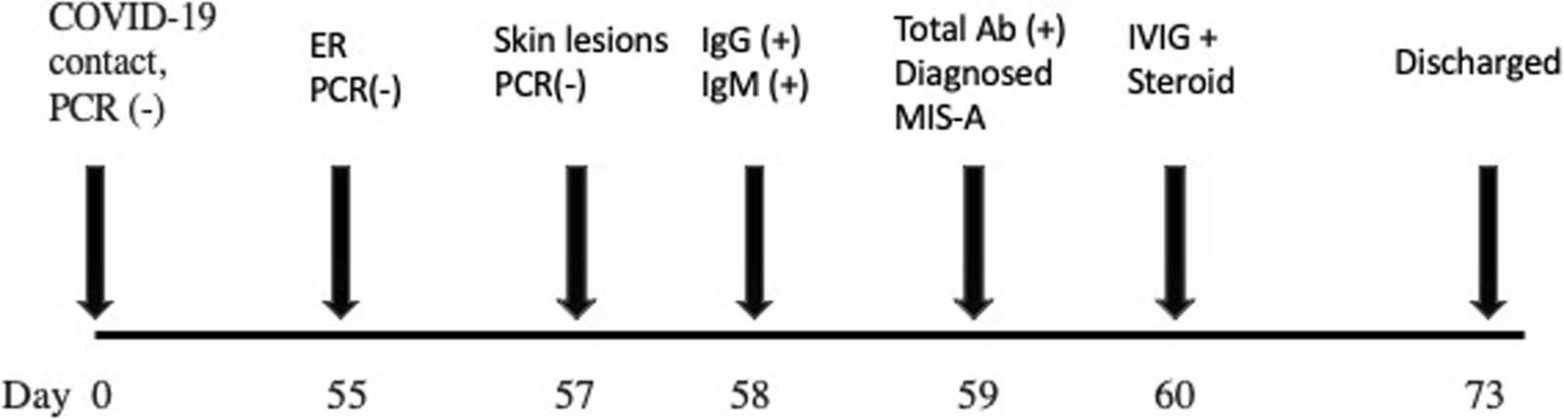
Showing chronologically tests and treatments done on the patient. *ER* Emergency room, *IVIG* Intravenous immunoglobulin

**Fig. 2:**
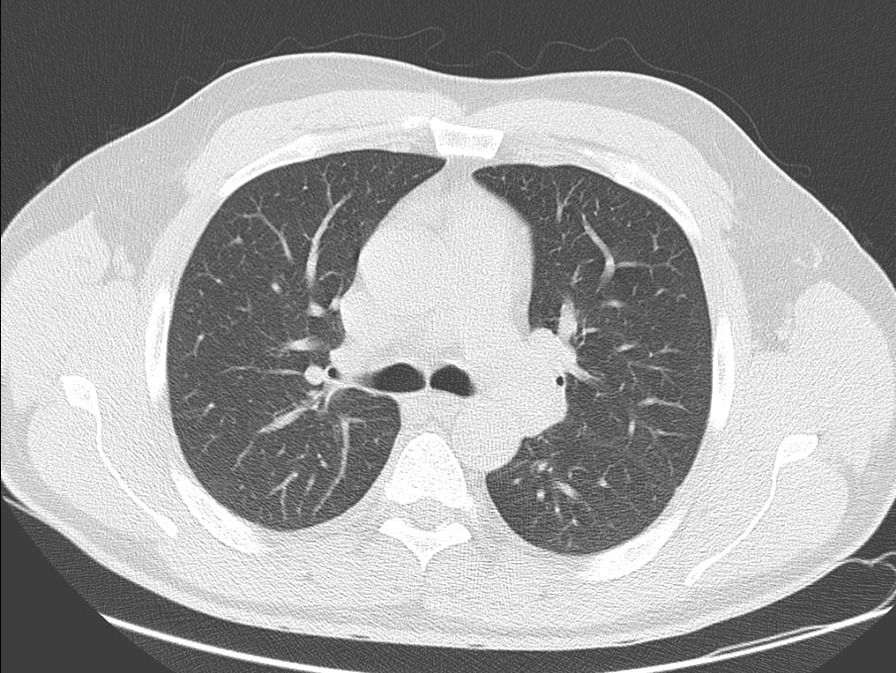
Showing the patient thorax CT on the day of hospitalization. CT revealed the patient's thorax was not involved by the disease

A 44-year-old male patient presented to the Emergency Department on Jun 07, 2021 (Day 55) with complaints of fever, fatigue, joint pain, nausea, headache, and redness of the eyes for 2 days. The patient reported no systemic disease and a close contact with his wife infected with COVID-19, but his SARS-CoV-2 PCR test was negative on RNA from a nasopharyngeal swab (Day 0). He developed a mild upper respiratory tract infection on his quarantine days between 13–24 April 2021. Once he was admitted by the emergency room (Day 55, Fig. 1), he showed normal oxygen saturation (SpO2: 99% at room air), an elevated fever (39 °C), tachycardia (102 pulses/min), arterial blood pressure (90/60 mmHg), redness in the eyes, and abdominal right lower quadrant tenderness. Elevated inflammatory markers, including ferritin (466 µg/L), C-reactive protein (CRP: 48 mg/L), D-dimer (2033 µg/L) were also found (Table 2). His transaminitis (aspartate aminotransferase: 96 U/L and alanine transaminase: 149 U/L) also increased (Table 2). Laboratory results showed white blood cells (6005 10^9/L), neutrophils (82.3%), lymphocyte (12.3%, 0.74 × 10^9/L), hemoglobin (13.4 gr/L), platelet (126,000 × 10^9/L), amylase (45U/L), lipase (37U/L), total bilirubin (1.39 mg/dl), prothrombin time (16.4″), and troponin-T (0.005 µg/L). His urine tests revealed elevated proteinuria (3+) and but not nitrite (-), erythrosine (-), and leukocyte (-). Additionally, his SARS-CoV-2 PCR test was found as negative during his ER stay (Fig. 1). Radiologically, it was not observed any pathological lesion in his abdominal and thorax CT (Fig. 2). Hepatosteatosis (grade 1) was, however, observed in his abdominal ultrasonographic examination. Electrocardiographic results showed negative T waves and nonspecific ST-T segmental changes in V1-V6 derivations. Echocardiography also showed a slight reduction of ejection fraction (EF, 60%) and mild mitral regurgitation (Table 3).

**Table 2:** The patient laboratory findings

Days	CRP mg/L	Ferritin ng/mL	D-dimer ng/mL	AST IU/L	ALT IU/L	Neut %	Lymph%	WBC cell/uL	Procal g/mL	Plt cell/uL
Day-0	48	466	2033	96	149	82.3	12.3	6	0.24	126
Trx-1	141	3978	1880	110	94	82.7	12.2	7.7	2.35	78
Trx -3	79	3419	964	162	155	82.2	14.2	11.7	0.85	167
Trx -5	29	1079	1120	121	197	54.8	36.3	7.3	0.21	288
Disch	3.2	424	435	404	663	65.2	55.2	5.2	0.065	265
Post-Op	1.3		400	27	31	46.8	40.6	4.3		253

Day 0 means the admission of the patient to the adult covid-19 service from the ER. IVIG and steroid treatment was given on day 1 (trx-1), day 3 (trx-3), day 5 (trx-5). The patient was discharged (Disch) on the day of the 14th, and the patient was seen day 18 (post-op) after the discharge. Neutrophil (Neut); Lymphocyte (Lymph); WBC (White blood cells); Procalcitonin (Procal); Platelet (Plt)

**Table 3:** The patient demographical, and laboratory outcomes during the hospital stay

Age (yrs), sex, race/ethnicity, location, BMI.	Underlying medical conditions	Clinical signs and symptoms	Previous respiratory illness/SARS-CoV-2 testing	SARS-CoV-2 testing at time of MIS-A admission	Imaging/Other diagnostic studies	Treatments	Outcome and length of stay	Screened microorganisms		Laboratory findings	
43-year old, male, caucasian , BMI:26 kg/m^2^	None	Fever, weakness, joint pain, abdominal pain, headache, redness eyes, skin lesions	Self isolation between Apr 13-24, 2021, COVID 19 PCR (-) on Apr 13, 2021	PCR (-) on Jun 7-9, 2021; Antibody test (+) on Jun 10 2021	Thorax CT: Normal; Abdominal CT:Normal; Abdominal ultrasonography: grade 1 hepatosteatos; at initial admission:[ECHO EF:60, ECG; negative T and non-specific ST-T segmental changes (V1-V6 )]At the discharge [ECHO: EF 64% and normal ECG.]	Steroid, IVIG, and LMWH	Disovered completely, and discharged at 14th of the treatment	*Multiple blood cultures*	N	*C-reactive protein( < 5 mg/L)*	144*
								Urine culture	N	*D-dimers ( < 500 µg/L)*	2033*
								*Rectal swab for multi-drug resistant bacteria*	N	*Ferritin (30-400 µg/L)*	3978*
								*HBV and HCV*	N	*Lactate dehydrogenase (135-225 U/L)*	589*
								*Leptospira*	N	*White blood count (4.5-10.5x10^9/L)*	11.7*
								*Borrelia burgdorferi*	N	*Neutrophil count ( 45-78 %)*	85.5*
								*SARS-CoV-2 (PCR)*	N	*Lymphocyte count (1.32-3.57x10^9/L)*	0.74**
								SARS-CoV-2 (Antibody test)	P	*Platelet count (150–400x10^9/L)*	78**
								*CMV IgG*	P	*Creatinine (07-1.2mg/dl)*	0.9*
								*CMV IgM*	N	*Troponin T(0.004 µg/L<)*	0.005*
								*EBV IgG*	N	*prokalsitonin(< 0.5 µg/L)*	2.35*
								*EBV IgM*	N	*AST(<40 U/L)*	404*
								*Weil felix test*	N	*ALT(<41 U/L)*	874*
								HIV	N	*T.Bilirubine(1.2mg/dl<)*	2.06*
								Rickettsia conorii	N	*Sedimantation(mm)*	61*
								HAV TOTAL	P	*Amilaz(28-100 U/L)*	60*
								HAV IgM	N	*Lipaz(13-60 U/L)*	55*
								Brucella	N	Protrombin time (11"-16.8")	16.4*
								TPHA	N		
								VDRL-RPR	N		

Screen microorganisms were checked from the patient urine, blood and rectal swab during his stay at the infectious department. Throughout his hospital stay, determined his maximum (*) and minimum (**) laboratory findings were shown

*BMI* Body mass index, *P* Positive, *N* Negative, *IVIG* Intravenous immune globulin, *LMWH* Low molecular weight heparin, * = Maximum value at the given range; ** = Minimum value at the given range

**Fig. 3:**
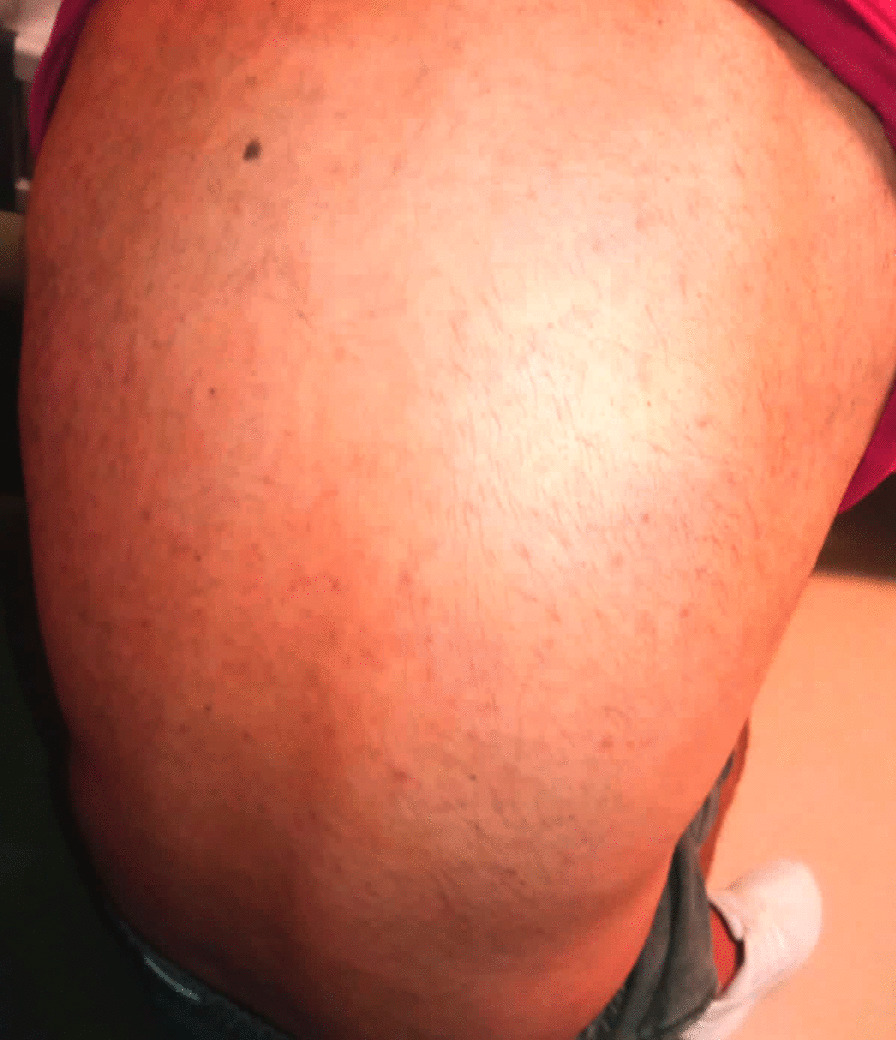
Skin lesions. The patient showed a maculopapular dermatological lesion in his most body

**Fig. 4:**
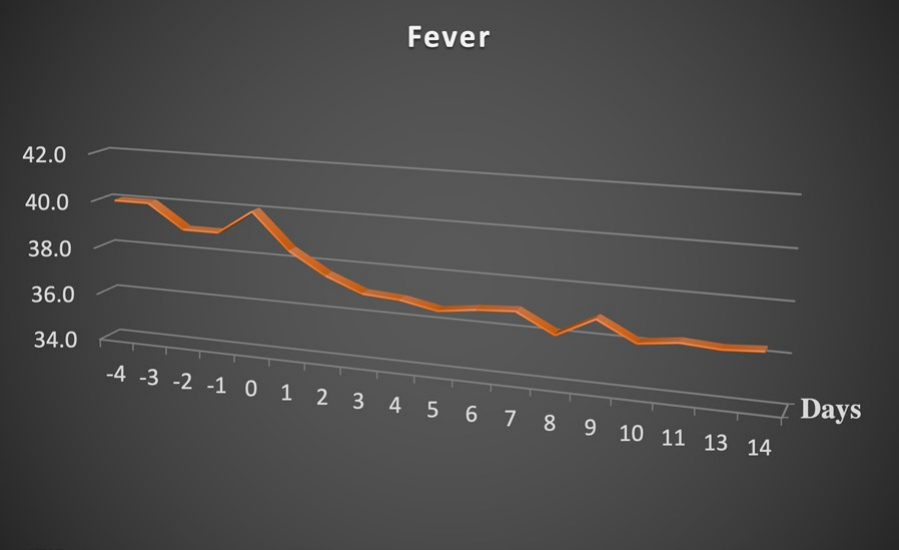
The effect of the treatment (steroid and IVIG) on fever. Fever was started dropping on the day of treatment (IVIG + steroid, day 0)

**Fig. 5:**
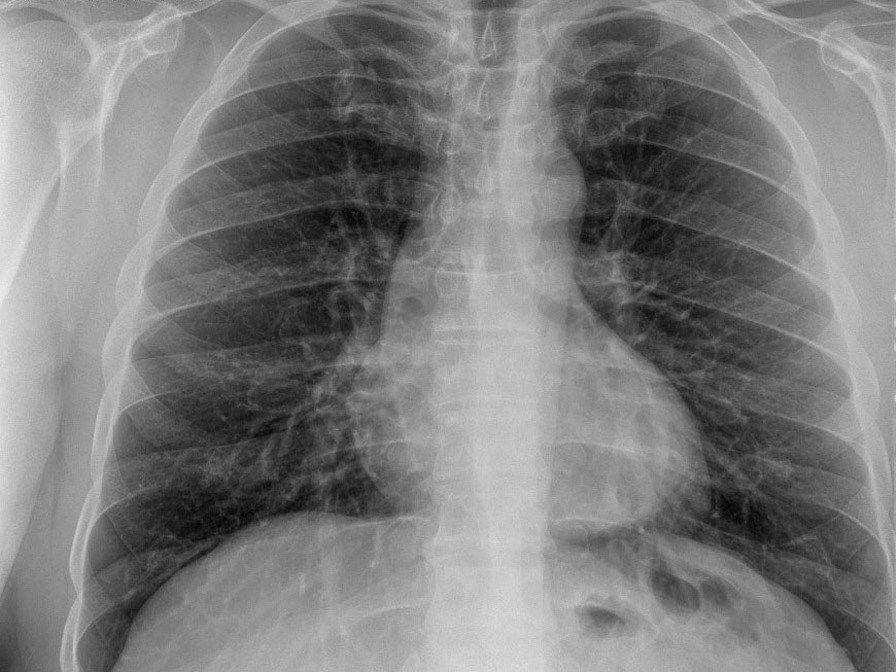
The patient pulmonary X-ray showing a normal appearance at the post-op (18 days after the discharge)

A general surgeon evaluated his nausea, elevated transaminase level, and right lower quadrant tenderness on abdominal examination to exclude his possible acute disease might be seen in the abdominal area. Then, the patient was referred to the department of infectious diseases to reveal an etiological reason for his unknown and uncontrolled fever. Cultures were made from his blood, urine, and stool. Meanwhile, the patient was given meropenem (1 gr, three times /day) and azithromycin (500 mg once/day). No pathogenic microorganism was found in his cultures (Table 3). On the second day of his hospitalization (Day 57), intense maculopapular pigmentation was observed in most of his body (Fig. 3). Following a dermatological consultation, azithromycin was discontinued and given doxycycline (100 mg twice/day). Even intravenous paracetamol (2 gr/day) was administered for six days to control his elevated fever, his fever remained at 39–40 °C. A broad-spectrum antibiotic therapy also did not improve the patient clinical and laboratory findings mentioned above. His COVID-19 PCR test was found negative at day 57 (Fig. 1). The patient was found positive for the IgM and IgG of COVID-19 using a rapid antibody test. The patient was transferred to the adult covid-19 service for a further treatment. Importantly, once the patient was admitted to the service, the patient's clinical condition had got worse than that of his initial laboratory findings (Table 2). Clinically, he showed high fever (39.5 °C), diffuse maculopapular rash, tachypnea (22 breaths/min), hypotension (90/58 mmHg), tachycardia (104 pulses/min), and SpO2 (98% at room air). A SARS Cov-2 total antibody test (Roche Cobas 8000), day 59, showed he had an increased level of response to COVID-19 (239 U/ml). Altogether so far, the clinical and laboratory findings share a striking resemblance to the newly defined MIS-A [2]. He was, then, given pulse methylprednisolone (day 1 and 2, 250 mg; day 3 and 4, 120 mg; day 5 and 6, 80 mg; day 7 and 8, 40 mg; day 9 and 10, 20 mg) and IVIG (20 mg/day, for 5 days) intravenously for 5 days after discontinuing meropenem (Fig. 1). Low molecular weight heparin (Clexane, 4000 anti-Xa/0.4 ml/day) was administered to avoid thromboembolism. The patient clinical conditions, including fever (Fig. 4), and other inflammatory markers dramatically were reduced on the second day of the treatment (Table 2). But liver enzymes have become normal 18 days after the discharge (Table 2). The patient history, including before and after the MIS-A diagnosis was summarized in Table 2. His vital signs were stable, and his skin lesions and the redness of his eyes were completely disappeared in the fourth day of the treatment. His cardiological findings, including ECHO and ECG, have become normal and the patient was discharged on day 73 (Fig. 1). The patient was seen on day 18 after the discharge. His laboratory (Table 2) and pulmonary X-ray (Fig. 5) showed he maintained his health.

## Discussion

Multisystem inflammatory syndrome is newly recognized clinical condition following SARS-CoV-2 infection. MIS-A patients might show unconfirmed COVID-19. Clinicians may, therefore, miss the diagnosis of MIS-A, MIS-A is also known to share several clinical findings with other diseases such as, septic shock [9]. They would make it difficult to diagnose, which resulting in delaying or missing an appropriate treatment. Therefore, it is crucial to distinguish MIS-C/A from its differential diagnoses. A comprehensive history, physical examination, laboratory investigation, especially the presence of severe pulmonary involvement and/or suspicious exposure history might help to diagnose MIS-A. To assess the risk of these diseases, water sources and exposure to animals should be questioned. Bacterial (such as staphylococci, streptococci, leptospirosis, and rickettsia) and viral infections can cause a toxic shock condition [9]. They, therefore, should also be considered in the differential diagnosis. Among common viral infections, viruses that can cause multisystem organ involvement, including enterovirus, adenovirus, parvovirus, enterovirus, rotavirus, and Epstein-Barr virus (EBV) should especially be kept in mind for the differential diagnosis [10]. Drug-induced hypersensitivity syndrome (DIHS) may need to be kept in mind [11].

Clinicians should, thus, consider MIS-A in adult patients showing unknown shock, heart failure, and/or gastrointestinal symptoms even in the absence of a confirmed history of COVID-19. It might have some similarities with Kawasaki Disease (KD), toxic shock syndrome (TSS), and macrophage activation syndrome (MAS) [12–14]. When MIS-C and KD disease are compared, the mean age of MIS-C is higher (mean age 8.5 and 3, in MIS-C and KD, respectively) [3, 15]. Fever and conjunctivitis are both present, and skin tissues are slightly more common. While gastrointestinal symptoms tend to predominate in MIS-C patients, traditional KD vomiting, and diarrhea are rare. MIS-C also has higher inflammatory markers (CRP, ferritin, and D-dimer) and more lymphopenia and thrombocytopenia. Cardiac involvement is more serious in MIS-C and high troponin, Pro-BNP, ECG changes are observed due to myocarditis. The frequency of hypotension and shock is higher. KD patients often have myocardial edema without ischemia. While the mortality rate in MIS-C is 2%, it is lower in KD at 0.17% [3].

In 2020, CDC reported 27 cases that met the diagnostic criteria and definition of MIS-A in its weekly mortality and morbidity weekly report (MMWR) [2]. In this report, the patient's age was between 21 and 50 years old [2]. Of the 16 patients, 12 had fever, 13 had gastrointestinal symptoms, 10 had ground-glass images. All these 16 patients showed an increased inflammatory reaction, cardiac involvement, and positive PCR or antibody test proving the presence of the previous Covid-19. No underlying medical conditions were reported in nine patients; six were obese, one had diabetes mellitus type 2, two had hypertension and one had obstructive sleep apnea. Eight (30%) of a total of 27 adults in this report had negative PCR and positive antibody testing, and most patients (24 out of 27) survived, but they were under intensive care settings [2]. In our case, cardiac involvement was mild, and there was pain and tenderness on deep palpation localized in the right lower quadrant mimicking acute appendicitis.

Recently, it has been reported MIS-A might give some dermatological manifestations, including a generalized erythematous, polymorphous rash with urticarial or and plaques with variable mucosal involvement; an erythematous, morbilliform eruption, facial swelling, and palmar desquamation [16–18]. Consistently, our patients showed diffuse maculopapular lesions, which started from the neck and extended to the ankles, and non-purulent conjunctivitis. So, dermatological findings can be a helpful clue to clinicians when diagnosing MIS-A [19, 20]. Consistently, the patient showed an intense maculopapular pigmentation between the neck and ankles on the second day of hospitalization. These lesions were most likely viral exanthems. They have started disappearing on the second day of the treatment and they were completely gone at 4-day. This kind of skin lesion would help in the diagnosis of suspected MIS-A cases.

Currently, only observational data are available to guide MIS-A treatment. Supportive care remains the mainstay of treatment. Although clinical trial data are lacking, many centers have described the use of immunomodulatory therapy (such as intravenous immune globulin [IVIG], corticosteroids, IL-1, and IL-6 inhibitors) [3]. American College of Rheumatology recommending IVIG and/or corticosteroids as first-line treatments and other biologic agents, including IL-1 (anakinra or canakinumab), IL-6 inhibitors (tocilizumab, sarilumab, or siltuximab), and TNF-α blocker (infliximab) might be given as second-line option [3, 21].

## Conclusion

This report indicated unknown fever and skin lesions would be a good sign for the diagnosis of MIS-A in patients who have COVID-19 contact history. Diagnostic tests (such as, antibody test) and treatment of MIS-A could result in reducing patient morbidity and mortality. The treatment that was provided in this case seemed to work nicely. However, there is a need for more study to accomplish for proper diagnostic findings (such as, early COVID-19 antibody test) and treatment approach. Regardless of the chosen treatment, it is plausible to start early treatment for the MIS-A to reduce patient morbidity and mortality.

## Data Availability

Not applicable.

## Abbreviations

ACE2: Angiotensin-Converting Enzyme 2
CDC: Centers for Disease Control
IVIG: Intravenous Immunoglobulin
KD: Kawasaki Disease
MAS: Macrophage Activation Syndrome
MIS: Multisystem Inflammatory Syndrome
MIS-A: Multisystem Inflammatory Syndrome in Adults
MIS-C: Multisystem Inflammatory Syndrome in Children
MMWR: Mortality and Morbidity Weekly Report
SARS-CoV-2: Severe Acute Respiratory Syndrome Coronavirus-2
TSS: Toxic Shock Syndrome
